# Dihydroartemisinin attenuates autoimmune thyroiditis by inhibiting the CXCR3/PI3K/AKT/NF-κB signaling pathway

**DOI:** 10.18632/oncotarget.22854

**Published:** 2017-12-01

**Authors:** Huijuan Liu, Qin Tian, Xiaoyu Ai, Yuan Qin, Zhanhong Cui, Meng Li, Jiahuan Yang, Denghui Zhai, Yanrong Liu, Shuang Chen, Jing Meng, Tao Sun, Honggang Zhou, Cheng Yang

**Affiliations:** ^1^ State Key Laboratory of Medicinal Chemical Biology and College of Pharmacy, College of Life Sciences, Nankai University, Tianjin, China; ^2^ College of Life Sciences, Nankai University, Tianjin, China; ^3^ Tianjin Key Laboratory of Molecular Drug Research, Tianjin International Joint Academy of Biomedicine, Tianjin, China

**Keywords:** DHA, AIT, CXCR3, PI3K, NF-κB

## Abstract

Dihydroartemisinin (DHA) is the first generation of naturally occurring artemisinin derivatives with antimalarial activity. Recent research showed that this drug also features immunosuppressive and anti-inflammatory properties. Autoimmune thyroiditis (AIT) is a common organ-specific autoimmune disease with no available effective drug treatment. In this study, we investigated effects of DHA on AIT *in vitro* and *in vivo*. Results showed that DHA can visibly reduce antithyroglobulin antibody and thyroid peroxidase antibody levels and regulate T helper cells (Th) 1/Th2 imbalance of experimental AIT mice. DHA also dose-dependently suppressed proliferation of lymphocytes induced by lipopolysaccharide and concanavalin A. DHA inhibited binding of C-X-C chemokine ligand 10 (CXCL10) and its receptor (C–X–C motif) receptor 3 (CXCR3), thus inhibiting calcium flow. DHA can also reduce expression levels of PI3-kinase (PI3K), p-PI3K, protein kinase B (AKT), p-AKT, nuclear factor (NF)-κB/p65, and p-NF-κB/p65. In conclusion, DHA may serve as treatment drug for AIT by inhibiting the CXCR3/PI3K/AKT/NF-kB signaling pathway.

## INTRODUCTION

Autoimmune thyroiditis (AIT) is a common organ-specific autoimmune disease and a type of autoimmune thyroid disease (AITD). Clinical types of AITD include Hashimoto’s thyroiditis (HT), Grave’s disease, and atrophic thyroiditis; many factors, such as genetic and environmental factors, are suspected to play important roles in AITD [1]. HT, which is chronic lymphocytic thyroiditis, is the most common clinical type with high prevalence of 13.4%. Prevalence of HT is high in middle-aged women, especially those in menopausal stage [2].

The most common manifestations of AIT comprise extensive lymphocyte infiltration, follicular collapse, and follicular cell degeneration [3]. Clinical features are characterized by enlargement of the thyroid gland with serum antithyroglobulin antibody (TGAb) and thyroid peroxidase antibody (TPOAb), of which TPOAb bears more importance [4]. Irregular treatment of patients will lead to increased thyroid autoantibodies, serious autoimmune inflammation damage to structure and function of thyroid follicular cells, and development of a series of diseases, such as hyperthyroidism, hypothyroidism, thyroid nodules, and thyroid cancer (TC) [5]. A significant association exists between HT and TC; statistics shows that incidence of TC reaches 45.7% in patients with HT and 29% in those without [6]. At present, primary treatment methods for AITD include thyroid hormone replacement therapy (only for patients with hypothyroidism), immunosuppressive therapy, and glucocorticoid therapy. These treatments pose different side effects. Therefore, novel and low-toxicity drugs should be developed for AITD treatment.

CXC chemokine ligand 10 (CXCL10) is an interferon (IFN)-γ-inducible chemokine that belongs to the ELR-CXC subfamily chemokine. CXCL10 exerts its function through binding to chemokine (C–X–C motif) receptor 3 (CXCR3), which is a seven-trans-membrane receptor coupled to G proteins. CXCL10 and CXCR3 play important roles in processes of AITD. Therefore, blocking CXCR3 may abrogate the related inflammatory process. CXCL10/CXCR3 may be considered a novel therapeutic target of thyroiditis [7–9].

Dihydroartemisinin (DHA) is the first generation of artemisinin derivatives with antimalarial activity that is 10 times higher than artemisinin. Studies showed that DHA exhibits anti-inflammatory and immunosuppressive effects with better efficiency and lower toxicity than artemisinin. Results of Youyou Tu’s group showed good curative effect of DHA treatment on systemic lupus erythematosus (SLE) [10]. And its clinical trials for SLE has been approved by SFDA in 2016. In this article, we investigate therapeutic effects of DHA on AIT and discuss its effect on CXCL10/CXCR3 signaling pathway.

## RESULTS

### DHA improves overall health of experimental AIT (EAT) mice and possesses immunity adjustment function

**Figure 1:**
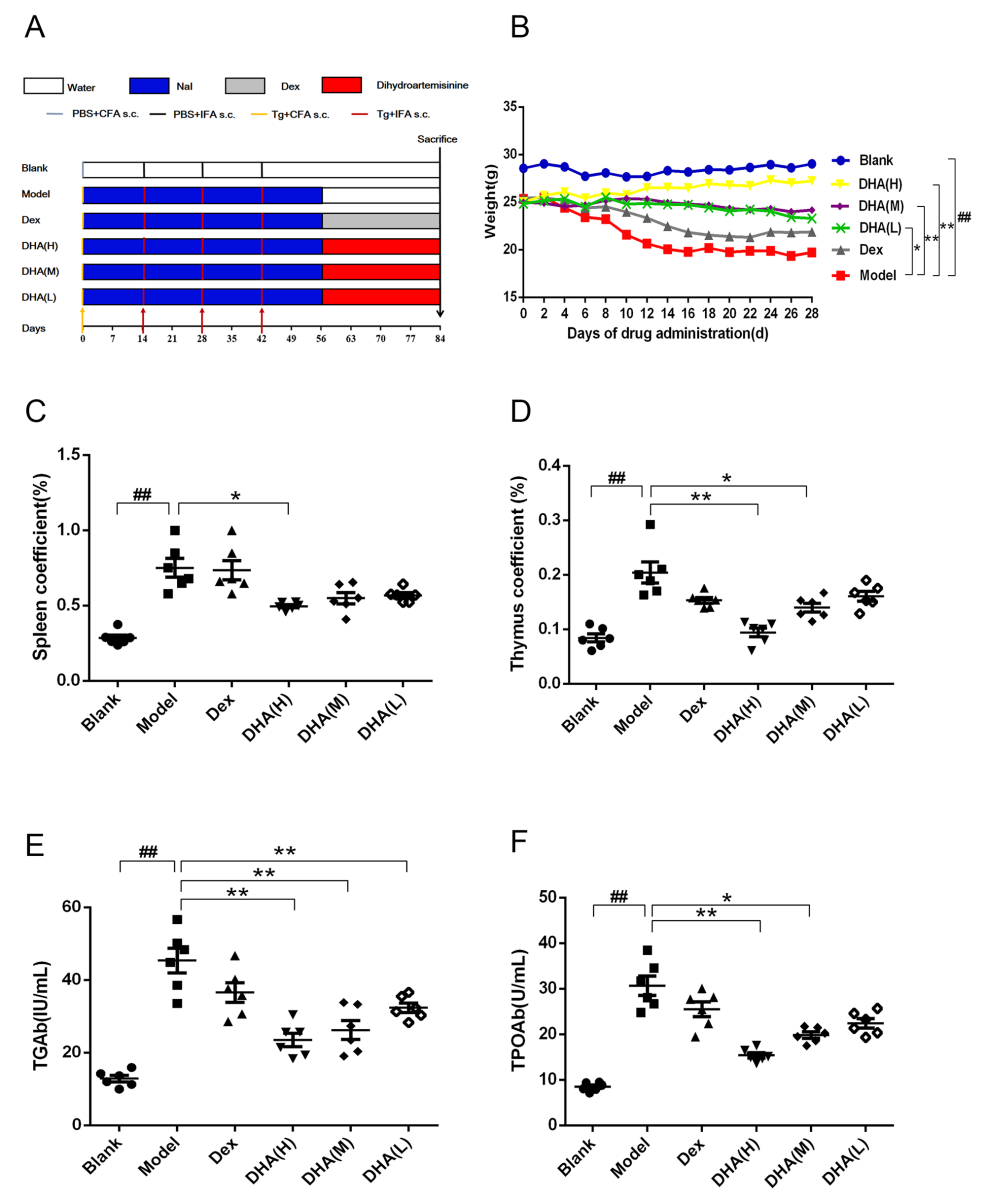
Effect of DHA on a C57BL/6J EAT mouse model **(A)** Workflow of animal experiment. **(B)** Body weights (g) of EAT mice with DHA administration. **(C and D)** Effects of DHA on spleen and thymus coefficients of EAT mice. **(E and F)** TGAb and TPOAb contents in sera of EAT mice after DHA treatment. Values are presented as mean ± SD. ^##^P < 0.01 versus control group. ^*^ P < 0.05, ^**^ P < 0.01 versus model group.

Figure 1A shows modeling and administration methods used in EAT mice. Weight of mice in all groups was monitored every other day until they were sacrificed. A significant difference was observed between body weight of the model and blank groups (Figure 1B). Body weight of mice in the model group decreased dramatically. A significant difference in body weight was detected between DHA treatment group and model group. DHA significantly improved body weight and survival state of EAT mice, whereas EAT mice in the Dex group did not present significant improvement in weight.

Thymus and spleen index of all DHA treatment groups markedly decreased compared with those of the model group (Figures 1C, 1D). By contrast, the positive drug dexamethasone can only reduce thymus coefficient. Results showed that TGAb and TPOAb levels in serum of the model group were significantly higher than those in the blank group and were remarkably reduced in DHA treatment groups (Figures 1E, 1F).

### DHA inhibits infiltration of inflammatory cells in the thyroid gland of EAT mice

**Figure 2:**
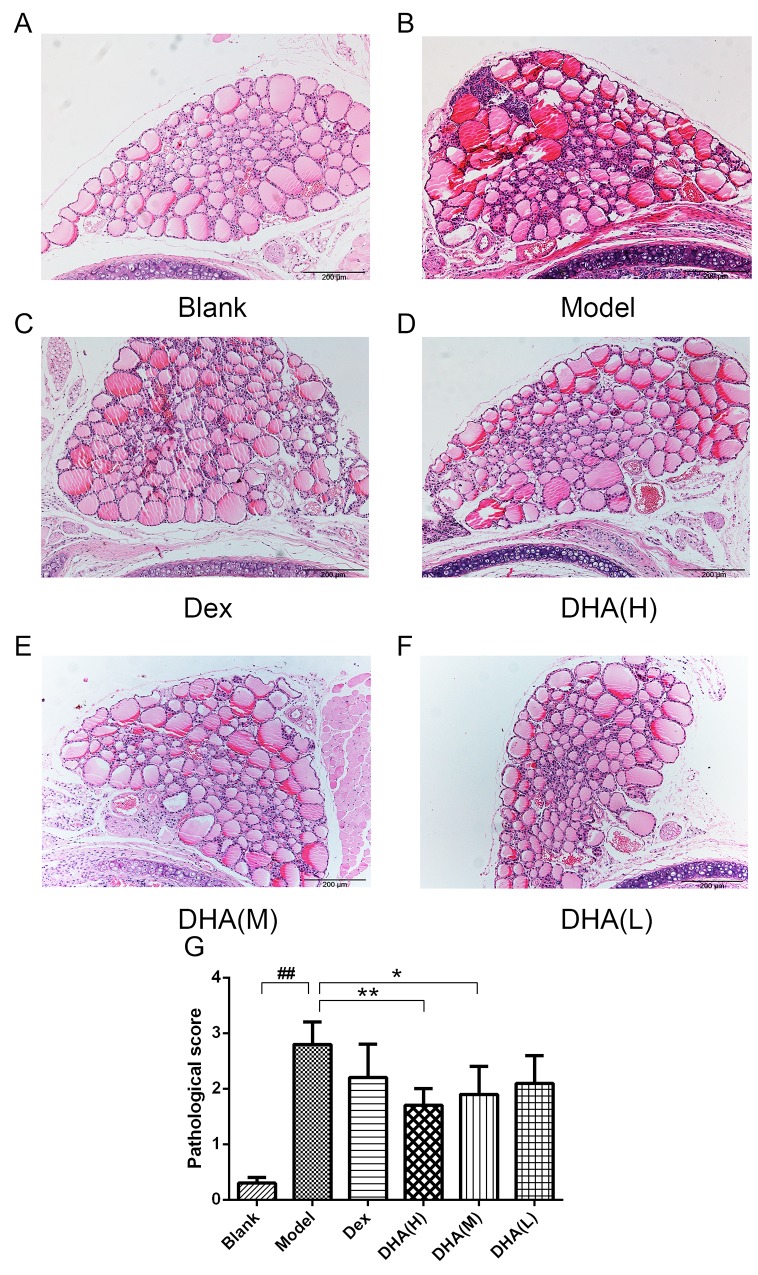
Histopathological changes in the thyroid gland of mice in experimental groups **(A)** Thyroid gland architecture of normal control mice (H&E, 100× magnification). **(B)** Tg- and NaI-induced EAT model mice showed a large number of inflammatory cell infiltrate (H&E, 100×). **(C)** Treatment with dexamethasone slightly inhibited infiltration of lymphocytes (H&E, 100×). **(D)** At 20 mg/kg/day, DHA significantly inhibited lymphocyte infiltration (H&E, 100×). **(E)** At 10 mg/kg/day, DHA can inhibit lymphocyte infiltration (H&E, 100×). **(F)** At 5 mg/kg/day, DHA slightly inhibited lymphocyte infiltration (H&E, 100×). **(G)** Analysis of areas of infiltration of inflammatory cells in experimental groups. Values are presented as mean ± SD. ^##^P < 0.01 versus control group. ^*^ P < 0.05, ^**^ P < 0.01 versus model group.

Various degrees of mononuclear cell infiltration were observed in EAT mice by hematoxylin and eosin (H&E) staining (Figures 2A–2F), with scores ranging from 0 to 4 (Figure 2G). Figure 2A shows a normal thyroid tissue without infiltration of inflammatory cells and thyroid follicle destruction. Thyroid follicle destruction caused by infiltration of inflammatory cells can be observed in most thyroid tissues in the model group (Figure 2B). Both DHA and Dex treatments can alleviate symptoms of inflammatory cell infiltration, with high dose and medium dose of DHA presenting a particular significance (Figures 2C–2F). Mean scores for mononuclear cell infiltration significantly reduced in DHA treatment groups (Figure 2G). This result indicates that DHA can reduce infiltration of intrathyroid mononuclear cells.

### DHA regulates Th1/Th2 cytokines

**Figure 3:**
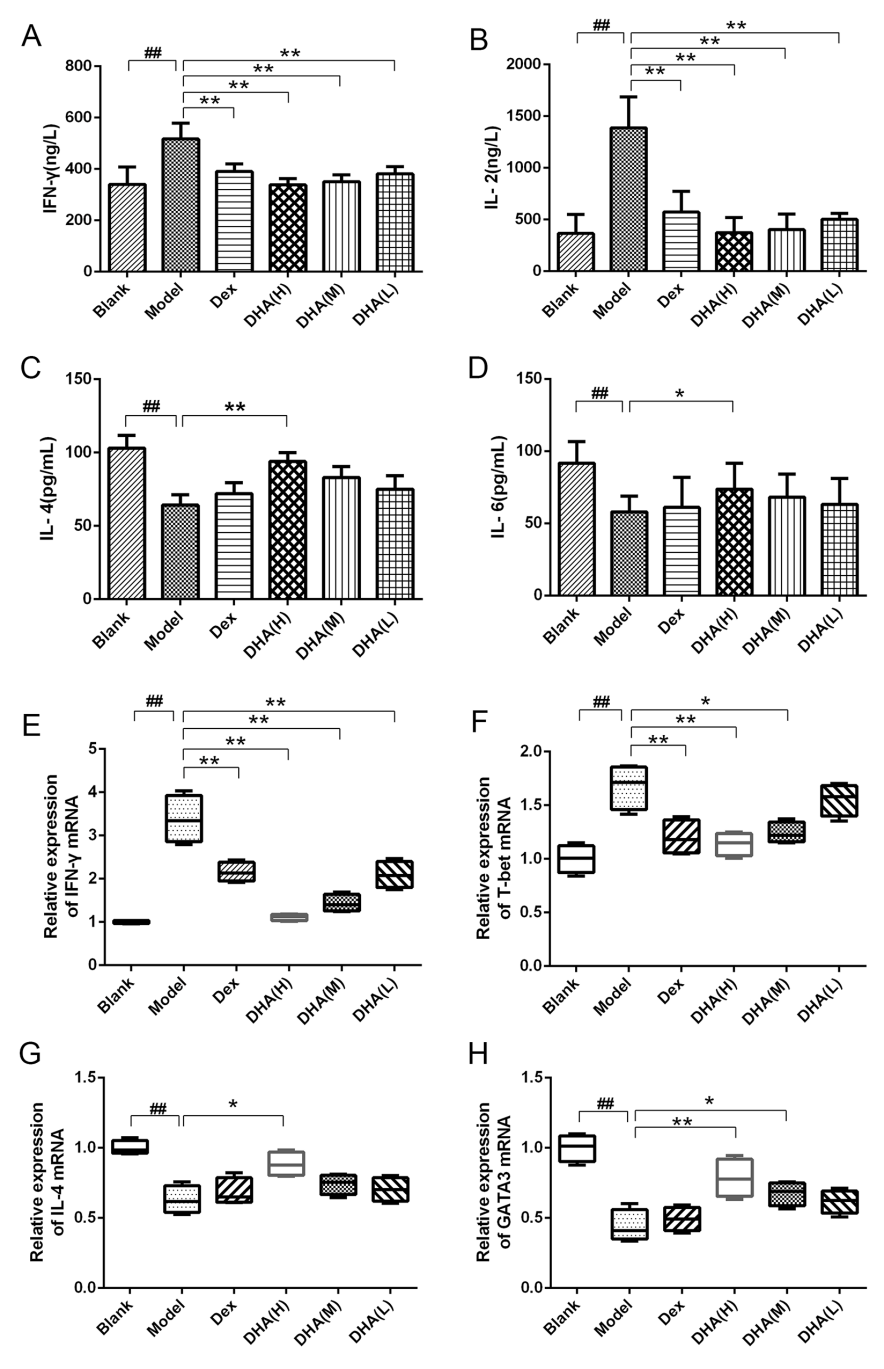
Effect of DHA on Th1/Th2 cytokine balance **(A and B)** Contents of Th1 type cytokines IFN-γ and IL-2 in sera of EAT mice. **(C and D)** Contents of Th2 type cytokines IL-4 and IL-6 in sera of EAT mice. **(E and F)** Relative mRNA expressions of Th1 typical cytokine IFN-γ and transcription factor T-bet in experimental groups. **(G and H)** Relative mRNA expression of Th2 typical cytokine IL-4 and transcription factor GATA3 in experimental groups. Values are presented as mean ± SD. ^##^P < 0.01 versus control group. ^*^ P < 0.05, ^**^ P < 0.01 versus model group.

Th1 type (IFN-γ and interleukin (IL)-2) and Th2 type (IL-4 and IL-6) cytokines were determined by enzyme-linked immunosorbent assay (ELISA). Contents of IFN-γ and IL-2 cytokines (Figure 3A, 3B) in sera of the model group increased significantly, whereas IL-4 and IL-6 cytokines decreased remarkably compared with that of the blank group (Figure 3C, 3D). DHA-treated group and Dex group showed significantly reduced contents of IFN-γ and IL-2 in serum of EAT mice (Figures 3A, 3B), whereas DHA H group exhibited significantly increased IL-4 and IL-6 contents compared with the model group (Figure 3C, 3D). This phenomenon indicates the imbalance between Th1 and Th2 in EAT mice, and this condition was alleviated by DHA treatment. The mRNA expressions of T subset-specific transcription factors and typical cytokines in the spleen were determined. As shown in Figures 3E–3F, relative mRNA expressions of Th1 typical cytokine IFN-γ and the transcription factor *T-box transcription factor 21*(T-bet) were significantly inhibited in DHA and Dex groups. High doses of DHA upregulated relative mRNA expression of IL-4 in the spleen of EAT mice (Figure 3G), and relative mRNA expression level of GATA-binding protein 3 (GATA3) was also significantly higher in the DHA-treated group (Figure 3H).

### DHA suppresses lymphocyte proliferation and chemotactic movement

**Figure 4:**
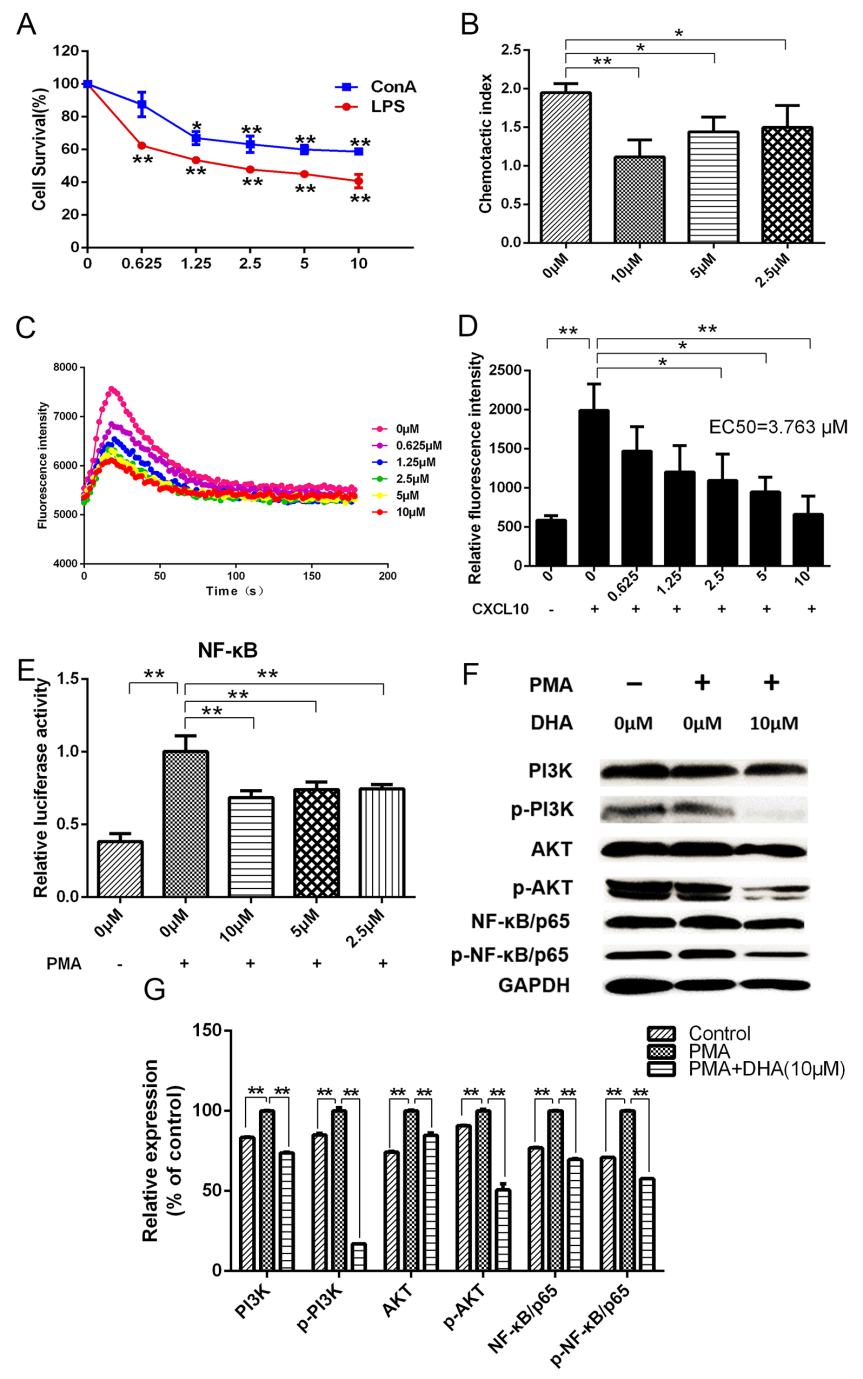
**(A)** Effects of DHA on lymphocyte proliferation. **(B)** Effects of DHA on chemotaxis index of lymphocytes. **(C and D)** Effects of DHA on calcium flow signals during binding of CXCL10 to CXCR3. **(E)** Dual-luciferase assay results for the effects of DHA on transcriptional activation activity of NF-κB in lymphocytes induced by PMA. **(F and G)** Western blot assay results for the effects of DHA on PI3K, p-PI3K, AKT, p-AKT, NF-κB/p65, and p-NF-κB/p65 expressions in lymphocytes induced by PMA.

DHA dose-dependently decreased proliferation of lymphocytes activated by lipopolysaccharide (LPS) and concanavalin A (Con A) (Figure 4A). Chemotactic movements of lymphocytes were compared by measuring their transmigration through transwell assay in response to different concentrations of DHA. Results showed that DHA significantly inhibited chemotaxis of lymphocytes (Figure 4B).

### DHA interrupts binding of chemokine CXCL10 to CXCR3 and inhibits the PI3-kinase (PI3K)/protein kinase B (AKT)/nuclear factor (NF)-kB pathway

The combination of chemokine CXCL10 and chemokine receptor CXCR3 can cause changes in intracellular calcium concentration. Results showed that DHA visibly decreased levels of calcium flow intensity and restrained binding of CXCL10 and CXCR3 (EC50=3.763 μM) (Figures 4C, 4D). Transcriptional activation activity of NF-kB/p65 in HEK293 cells was inhibited by DHA according to results of dual-luciferase assay (Figure 4E). Phorbol-12-myristate-13-acetate (PMA) promoted protein expression of lymphocytes according to Western blot. Compared with the group induced by PMA, expression of CXCR3 downstream proteins, such as PI3K, p-PI3K, AKT, p-AKT, NF-kB/p65, and p-NF-kB/p65, were significantly reduced by DHA (Figures 4F, 4G).

### DHA regulates immune and inflammatory signaling pathways

**Figure 5:**
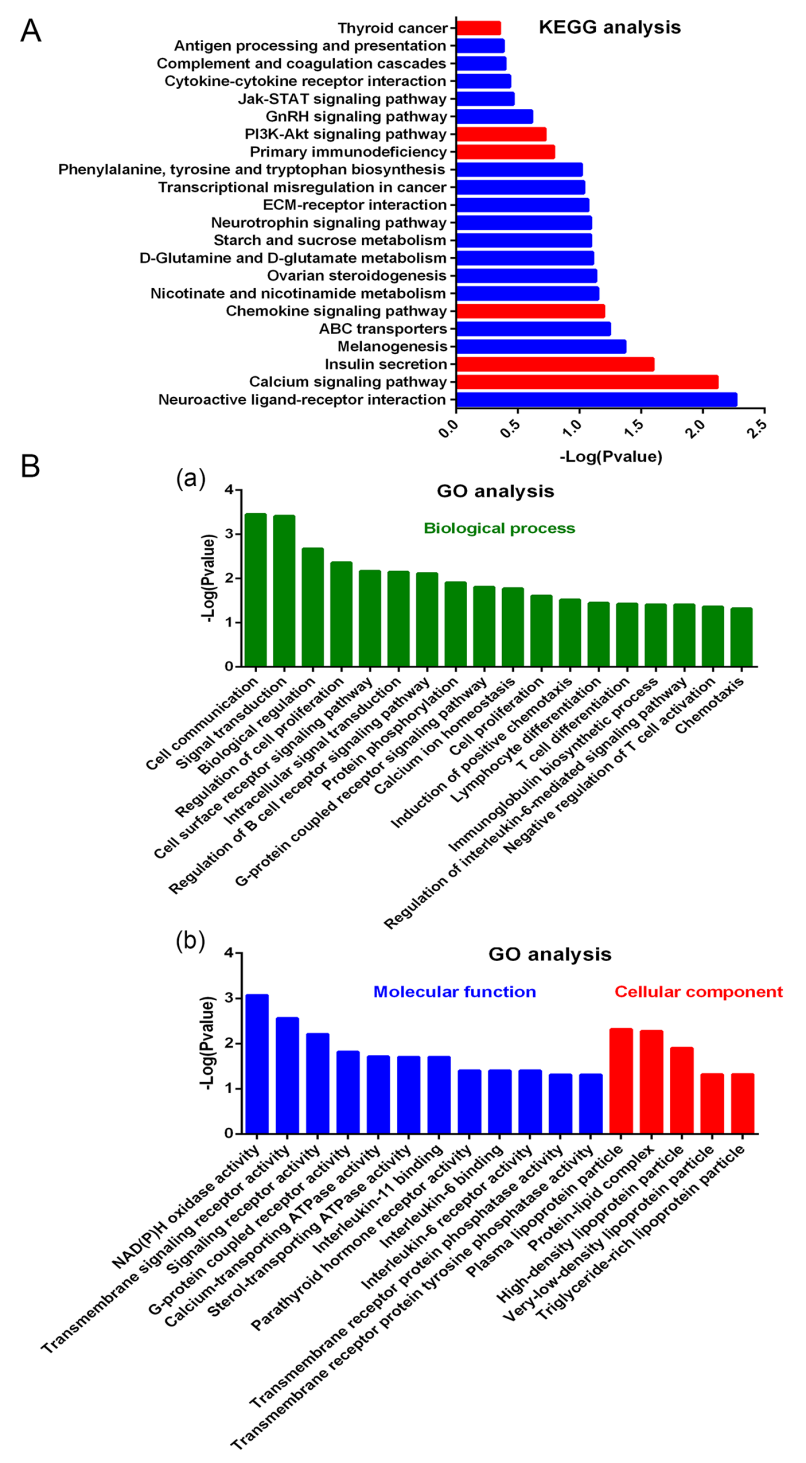
Effects of DHA on lymphocyte expression detected by microarray **(A)** KEGG analysis for effects of DHA on signal pathways. **(B)** GO analysis for the effects of DHA on biological process **(a)**, molecular function, and cellular component **(b)**.

**Figure 6:**
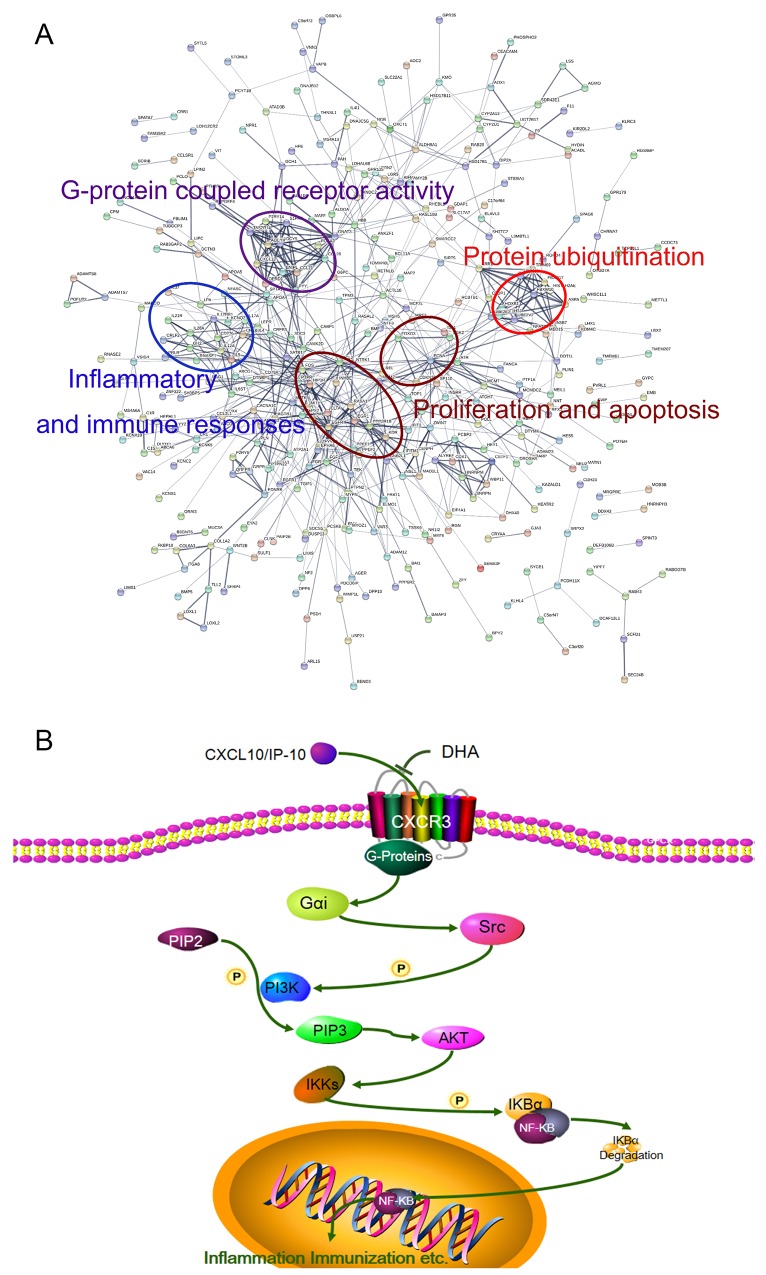
**(A)** Protein–protein interaction networks of differentially expressed proteins. Significantly changed proteins correlated with G protein-coupled receptor activity, inflammation and immune responses, proliferation and apoptosis, and protein ubiquitination. **(B)** DHA signaling pathway in treatment of AIT.

As observed from the Kyoto Encyclopedia of Genes and Genomes (KEGG) analysis (Figure 5A), DHA affects a large number of signal pathways, such as calcium signaling pathway, chemokine signaling pathway, primary immunodeficiency, PI3K/AKT signaling pathway, and TC. Gene Ontology (GO) analysis results showed the mainly influenced functions in biological processes, molecular functions, and cellular components. As shown in Figure 5A, biological processes, such as cell communication, signal transduction, lymphocyte differentiation, and chemotaxis, were notably influenced by DHA. Figure 5B shows molecular function and cellular components, such as G-protein-coupled receptor activity, plasma lipoprotein particle, IL-6 binding, and IL-6 receptor activity, that are affected by DHA. Further analysis revealed that the modified proteins are mainly correlated with G protein-coupled receptor activity, inflammation and immune responses, proliferation and apoptosis, and protein ubiquitination (Figure 6A).

## DISCUSSION

Incidence of AIT in female is significantly higher than that in male [11]. EAT induced by thyroglobulin (Tg) immunization and NaI is a classical and widely used animal model in studies of AIT pathogenesis. Using the EAT mouse model, the current study investigated therapeutic effects of DHA on AITD and its potential mechanisms. In this study, overall health of EAT mice improved after administration of DHA, and extent of mononuclear cell infiltration in the thyroid was significantly lower in DHA treatment group in comparison with that of model group. Specifically, blood analysis also showed remarkably lower TGAb and TPOAb levels in the DHA treatment group than in the model group. Weights of spleen and thymus serve as important indices of immune function [12]. Our findings indicated that spleen and thymus coefficients decreased significantly in a dose-dependent manner in DHA-treated mice, suggesting that DHA probably performs immunosuppressive functions. These results indicate that treatment with DHA improved the condition of EAT mice by inhibiting immune and lymphocyte infiltration.

Based on their respective patterns of cytokine production, CD4+ (Th) cells consist of at least two different subsets, namely, Th1 and Th2. Th1 cells typically produce cytokines, such as IFN-γ and IL-2, which are responsible for cell-mediated immune function protects cells against intracellular microbes. Th2 cells produce IL-4 and IL-6, which participate in atopic and allergic reactions [13]. T-bet and GATA-3 are two major Th-specific transcription factors that regulate expressions of Th1 or Th2 cytokine genes and play important roles in differentiation of Th cells [14]. Th1/Th2 imbalance plays a vital role in occurrence and development of AIT [15]. In this study, imbalance in Th1 and Th2 in EAT mice was improved by treatment with DHA. Therefore, DHA may partially alleviate AIT through modulation of Th1/Th2.

CXCL10 is a chemokine induced by IFN-γ, and it has been proposed as an inflammation marker of AIT [7–9]. CXCL10 may be a marker of a more powerful and more aggressive inflammatory response in the thyroid or cause destruction of thyroid tissues and hypothyroidism [16]. CXCL10 powerfully recruits lymphocytes expressing CXCR3 in inflamed tissues. Binding of CXCL10 chemokines and CXCR3 plays a vital role in Th immune response, which is responsible for organ-specific autoimmune diseases [7]. In this study, as observed through calcium flow experiments, DHA significantly inhibited interaction of CXCL10 and CXCR3.

NF-kB plays a vital role in inflammatory responses and immune reactions [17]. Inhibition of PI3K/AKT signaling pathway provides a new therapy for autoimmune diseases [18]; PI3K/AKT performs an important role in signal activation of NF-kB [18, 19]. Our results showed that DHA can inhibit transcriptional activation activity of NF-kB. Western blot results revealed that DHA inhibited expression of p-NF-kB by dramatically inhibiting phosphorylation of PI3K/AKT. Activation of CXCR3 increased activity of PI3K and its downstream pathway [20]. Based on the above experimental results, we hypothesize that DHA can inhibit CXCL10 binding to CXCR3, thereby inhibiting downstream signaling pathways of PI3K/AKT/NF-kB, which execute important roles in AIT.

## MATERIALS AND METHODS

### Mice and experimental protocol

Six-week-oldfemale C57BL/6J mice weighing 20±2 g were purchased from the Animal Center of the Academy of Military Medical Sciences (Beijing, China) and maintained in the Tianjin International Joint Academy of Biomedicine under specific pathogen-free conditions. All protocols conformed to Animal Ethics Committee of the Tianjin International Joint Academy of Biotechnology and Medicine.

### Chemicals and reagents

DHA and sodium iodide were purchased from J&K Scientific Ltd (Beijing, China). Porcine Tg, LPS, con A, complete Freund’s adjuvant (CFA), and incomplete Freund’s (IFA) adjuvant were provided by Sigma–Aldrich Co. Ltd (St. Louis, MO, USA). PMA was purchased from Beyotime Institute of Biotechnology (Jiangsu, China). IP-10 (CXCL10) was obtained from PeproTech EC Ltd. (Rocky Hill, NJ). CXCR3 plasmid was purchased from GeneCopoeia Inc (Rockville, MD, USA). FLIPR Calcium 6 Assay Kit was provided by Molecular Devices, LLC (Sunnyvale, CA). PI3K antibody, p-PI3K antibody, AKT antibody, p-AKT antibody, NF-kB/p65 antibody, p-NF-kB/p65 antibody, and CXCR3 antibody were purchased from Affinity Bioreagents Co. Ltd (Colorado, USA). Secondary antibodies were purchased from EarthOx (San Francisco, USA).

### *In vivo* study

Female C57BL/6J mice were immunized using porcine Tg as immunogen with Freund’s as adjuvant. Porcine Tg (2 mg/ml) was dissolved in phosphate-buffered saline (PBS) and emulsified 1:1 in CFA. Mice were immunized by subcutaneous injection of Tg (0.1 ml, s.c.) after emulsification on day 0. A second subcutaneous injection was administered on day 14 using the same amount of Tg in IFA and once every two weeks for a total of six weeks. The blank group received subcutaneous injection of similar amounts of PBS and CFA or IFA at the same days [21, 22].

After immunization, C57BL/6J mice were randomly divided into six groups (*n*=6 each): blank group (physiological saline; *n*=6), model group (physiological saline; *n*=6), Dex group (4.5 mg/kg of dexamethasone; *n*=6), DHA (H) group (20 mg/kg of DHA; *n*=6), DHA (M) group (10 mg/kg of DHA; *n*=6) DHA (L) group (5 mg/kg of DHA; *n*=6). Dexamethasone and DHA were administrated by oral gavage once a day for 28 days to the treatment groups. The blank and model groups received the same volumes of physiological saline. All mice were weighed once every two days and sacrificed after four weeks of treatment.

### Spleen and thymus coefficients of mice

Spleens and thymuses were removed from mice and weighed to obtain coefficients of spleen and thymus. Spleen index (mg/g) = spleen weight/body weight, and thymus index (mg/g) = thymus weight/body weight.

### Thyroid histology

Thyroid tissues were removed and were fixed in 10% formalin in PBS for at least 24 h and stained by H&E. Grading was performed blindly to the experimental groups from which tissues originated. Thyroids that exhibited inflammatory cell infiltration were considered cases of thyroiditis and subsequently subjected to H&E staining, whereas the presence of mononuclear cell infiltration was scored as follows: 1) interstitial accumulation of inflammatory cells distributed between two or more follicles; 2) one or two foci of inflammatory cells reaching at least the size of one follicle; 3) 10% to 40% of the thyroid replaced by inflammatory cells; 4) > 40% of the thyroid replaced by inflammatory cells. Mean grades of EAT were assigned as follows: 0 to 1, negative; 1 to 2, mild; 2 to 3, severe; and 3 to 4, acute [23].

### ELISA

Blood samples were harvested from the orbit of each mouse at day 28 post-treatment to measure contents of serum IFN-γ, IL-2, IL-4, IL-6, TPOAb, and TGAb by using ELISA Kit (Mlbio, Shanghai, China). All tests were conducted according to manufacturer’s instructions.

### Assay of lymphocyte proliferation

Mice were sacrificed, and the spleen was aseptically separated. Spleens were separated into individual cells and filtered with a 40 μm-pore size cell strainer. Red blood cells were removed from cell suspensions by incubation for 5 min at room temperature in red cell lysis buffer and subsequently washed twice with PBS. Splenocytes were diluted in RPMI 1640 medium with 10% newborn bovine serum to a final concentration of 5×10^6^ cells/mL. Spleen cells added with LPS 20 μg/mL or con A 20 μg/mL were seeded into a 96-well plate and incubated at 37°C and 5% CO_2_. After overnight incubation, cells were treated with various concentrations of DHA. After incubation for 72 h, cell viability was measured after addition of 25 μL 3-(4,5-dimethythiazol-2-yl)-2,5-diphenyltetrazolium bromide(MTT) at 37°C for 4 h, and then 150 μL dimethylsulfoxide was added to dissolve formazan crystals [24]. Absorbance of each well was measured at 570 nm using a microplate reader (Multiskan™ FC, Thermo Fisher Scientifi, Waltham, MA, USA).

### Reverse transcription polymerase chain reaction (RT-PCR)

Fresh spleens were removed from mice, rapidly frozen in liquid nitrogen, and then stored at −80°C prior to experiments. TRNzol Universal Reagent (TIANGEN BIOTECH, Beijing, China) was used to extract total RNA and for reverse transcription into cDNA after quantification by Nanodrop 2000 (Thermo Fisher Scientific, Wilmington, DE, USA). Primers were synthesized by AuGCT (Table 1). Quantitative PCR (Q-PCR) was performed using the Applied Biosystems 7900 Fast Real-Time PCR system (Applied Biosystems, Foster City, California, USA). Results were calculated using ΔCT method, and ratios of all target genes in each group were based on their relation expression versus the level of GAPDH gene expression [25].

**Table 1:** Primer sequences for Q-PCR

Gene	Primers (5′-3′)
IFN-γ	5′-CGGCACAGTCATTGAAAGCCTA-3′
	5′-GTTGCTGATGGCCTGATTGTC-3′
T-bet	5′-GTTCAACCAGCACCAGACAGAG-3′
	5′-TGGTCCACCAAGACCACATC-3′
IL-4	5′-ACGGAGATGGATGTGCCAAAC-3′
	5′-AGCACCTTGGAAGCCCTCAGA-3′
GATA3	5′-GGATGTAAGTCGAGGCCCAAG-3′
	5′-ATTGCAAAGGTAGTGCCCGGTA-3′
GAPDH	5′-ACTCCACTCACGGCAAATTC-3′
	5′-TCTCCATGGTGGTGAAGACA-3′

### Transient transfection and dual-luciferase assay

Human embryonal kidney (HEK)-293 cells were obtained from KeyGen Biotech (Nanjing, China) and grown in DMEM with 10% fetal bovine serum at 37°C in 5% CO_2_. Cells were plated in 96-well plates at a concentration of 2×10^4^ cells/well, and Lipofectamine 2000 transfection reagent was used for transfection. Recombinant plasmids NF-kB and TK were cotransfected into HEK-293 cells for 6 h. Cells were cultured with various concentrations of DHA and/or 30 nM PMA for 24 h, and relative luciferase activity in cell extract was determined. Firefly luciferase activity was measured using ONE-Glo^TM^ Luciferase Assay System (Promega Corp. Madison, WI) according to manufacturer’s protocol [26].

### Intracellular Ca^2+^ concentration measurements

HEK-293 cells stably transfected with CXCR3 were seeded into blank-walled 384-well plates. Cells were incubated for 12 h to allow plate seeding. Calcium 6 kit was used for dye loading. After cell attachment, culture medium was replaced with another culture medium containing different concentrations of DHA incubated with dye for 2 h. A multifunctional microplate detecting instrument (Tecan Spark™ 10M) was used to add CXCL10 chemokines (100 ng/ml) and to evaluate changes in florescence. Ca^2+^ concentrations were calculated after determining maximum and minimum ratios of fluorescence in the presence and absence of saturation levels of calcium [27].

### Chemotaxis assay

Lymphocyte migration was evaluated by chemotaxis assays through a 5 μm pore polycarbonate filter in 24-well transwell chambers (Corning Costar, Cambridge MA). Lymphocytes were separated from spleens. Cell suspensions with various concentrations of DHA were placed in the upper chambers, whereas CXCL10 chemokines were added to the bottom chamber of the transwell (0.6 mL). The plate was incubated at 37°C in 5% CO_2_ atmosphere for 1 h. Results are shown as chemotactic index which was calculated by dividing the percentage of cells migrated in the presence of CXCL10 by the percentage of cells migrated in its absence [27, 28].

### Western blot

Lymphocytes induced by PMA (30 nM) were treated with 0, 5, and 10 μM DHA for 48 h. Proteins of harvested cells were extracted using RIPA buffer (Beyotime, Jiangsu, China). Proteins were separated by electrophoresis and transferred onto polyvinylidene difluoride membranes (Millipore, Bedford, MA, USA). Membranes were blocked and incubated with primary antibodies (PI3K, p-PI3K, AKT, p-AKT, NF-kB/p65, and p-NF-kB/p65). GAPDH was used as loading control. After incubation with respective alkaline phosphatase-conjugated secondary antibodies (Santa Cruz, CA, USA), proteins were detected by an enhanced chemiluminescence Western blot kit (Amersham Corp., Buckinghamshire, United Kingdom) and exposed to a chemiluminescent film. Relative band intensity was quantified using Image J software (U.S. National Institutes of Health, Bethesda, MD, USA).

### Microarray

Lymphocytes induced by PMA (30 nM) were untreated or treated with DHA for 24 h. Cells were then washed thrice with PBS and treated with TRNzol Universal Reagent. Samples were sent to GENERGY Biotechnology (Shanghai) Co., Ltd. for expression microarray detection. GO analysis was used to detect DHA-induced changes in molecular functions, biological processes, and cellular components. STRING database was used to analyze protein–protein interactions, and KEGG analysis revealed inhibition of various signaling pathways.

### Statistical analyses

All data were expressed as means ± standard deviation (SD). Comparisons between groups were performed by one-way analysis of variance followed by Bonferroni post hoc test (SPSS software package version 17.0, SPSS Inc., Chicago, IL, USA). Level of significance was set at P < 0.05.
